# Proteome-wide identification and functional analysis of ubiquitinated proteins in peach leaves

**DOI:** 10.1038/s41598-020-59342-3

**Published:** 2020-02-12

**Authors:** Yanbo Song, Xiaojing Shi, Yanli Zou, Juanru Guo, Nan Huo, Shuangjian Chen, Chengping Zhao, Hong Li, Guoliang Wu, Yong Peng

**Affiliations:** 1 Life Science College, Shanxi Agricultural University, Taigu, Shanxi 030801 PR China; 2 Institute of Pomology, Shanxi Academy of Agricultural Sciences, Taigu, Shanxi 030801 PR China; 3 Horticulture College, Henan Agricultural University, Zhengzhou, Henan 450002 PR China; 4 Shanghai Applied Protein Technology Co., Ltd, Shanghai, 201100 PR China

**Keywords:** Biochemistry, Plant sciences

## Abstract

Ubiquitination is a critical post-translational modification machinery that governs a wide range of cellular functions by regulating protein homeostasis. Identification of ubiquitinated proteins and lysine residues can help researchers better understand the physiological roles of ubiquitin modification in different biological systems. In this study, we report the first comprehensive analysis of the peach ubiquitome by liquid chromatography-tandem mass spectrometry-based diglycine remnant affinity proteomics. Our systematic profiling revealed a total of 544 ubiquitination sites on a total of 352 protein substrates. Protein annotation and functional analysis suggested that ubiquitination is involved in modulating a variety of essential cellular and physiological processes in peach, including but not limited to carbon metabolism, histone assembly, translation and vesicular trafficking. Our results could facilitate future studies on how ubiquitination regulates the agricultural traits of different peach cultivars and other crop species.

## Introduction

Peach is a highly popular and well-recognized fruit first cultivated in China during ancient times. It is considered an excellent source of vitamin C, niacin, potassium and dietary fiber. As a result, the recent global trend of healthy eating has greatly stimulated the demand for peach and the related food products. As of 2014, the combined area of peach orchards in China is estimated at around 7,260 square kilometers, with a total production of 12.4 million tons^1^. The long history of peach cultivation has resulted in hundreds of distinct cultivars. Peach growers have spent decades in a continuous quest to enhance the quality of different peach cultivars through crossbreeding. Recently, biotechnology-facilitated engineering has shown enormous promise in rapidly generating new peach varieties with desirable agricultural traits. Still, further accomplishments would depend on improved understanding of the diverse molecular pathways in peach and their connections to its physiology.

Ubiquitination is one of the most critical post-translational modification mechanisms with a wide range of cellular functions that center on the control of protein load^2,3^. Ubiquitin is a small but abundantly present protein consisting of 76 amino acids with a C-terminal diglycine tail. The ubiquitination pathway is essentially an enzymatic cascade involving the cooperative action of three enzymes, including the E1, E2 and E3 ligases^2,4^. During this process, the C-terminal carboxyl group of ubiquitin is first adenylated by E1 in the presence of ATP and then forms a thioester linkage with the catalytic cysteine residue of the ligase^5^. The E1 then binds a second ubiquitin with its adenylation domain and recruits its cognate E2.This triggers a transthioesterification reaction where the E1-conjugated ubiquitin unit is transferred to the catalytic cysteine of the E2^6^. Finally, an E3 is involved in the transfer of ubiquitin from E2 to the ε-amino group of one or more lysine residues in the protein substrate^7^. E3 ligases can largely be classified, based on the signature domain that they contain, into homologous to the E6AP carboxyl terminus (HECT) E3s, really interesting new gene (RING) E3s and RING-related E3s^8^. The HECT domain is capable of forming a covalent thioester intermediate with ubiquitin as part of its catalytic transfer from E2 to the target substrate^9^. In comparison, RING and RING-related E3s, such as members of the U-box family, mediate the direct transfer of ubiquitin from E2 to the substrate primarily by acting as a molecular scaffold between the two^10^. The biological function of ubiquitination varies greatly according to factors such as the number of ubiquitin units attached, the conjugation pattern of the polyubiquitin chain, and the cellular context^11–13^. For example, proteins with a lysine 48-, lysine 29- or heteroltypic lysine 11-linked polyubiquitin chain are targeted for proteasomal degradation^14,15^. Modification that occurs on other sites, such as lysine 6, 11 or 63, could play other biological roles, including but not limited to translation, endocytic trafficking and inflammatory response^14,16^.

Not surprisingly, the ubiquitin-proteasome system (UPS) is also heavily involved in every aspect of plant physiology, including but not limited to embryogenesis, growth, reproduction and abiotic stress^17^. Recently, the rapid advances in mass spectrometry (MS)-based proteomic approaches have allowed researchers to systematically analyze ubiquitination sites in plant proteins and unearth previously unknown ubiquitin-associated regulatory mechanisms. Using a combination of immune affinity purification techniques and high-resolution liquid chromatography (LC)-tandem MS/MS, Xie and coworkers reported the first rice ubiquitome consisting of 861 ubiquitination patterns on a total of 464 proteins^18^. In another study, comprehensive profiling of the wheat ubiquitome revealed 433 lysine modification sites in 285 proteins, modulating cellular activities such as protein translation, carbohydrate metabolism, cell signaling transduction, etc.^19^. The ubiquitination targets identified from these studies no doubt provided a useful starting point for further investigating the implications of ubiquitination in various plant physiological processes.

In this study, we report the first peach ubiquitome by employing an LC-MS/MS approach. In total, 544 ubiquitination sites on a total of 352 protein substrates were identified. Functional analysis suggested that ubiquitination could be involved in regulating not only essential metabolic pathways but also key cellular functions such as histone assembly, translation and vesicular trafficking. These findings could allow agricultural researchers to better understand the roles of ubiquitination in peach physiology and identify novel ubiquitin-driven regulatory mechanisms that could be exploited for peach improvement.

## Results

### Overview of the ubiquitomic analysis results

**Figure 1:**
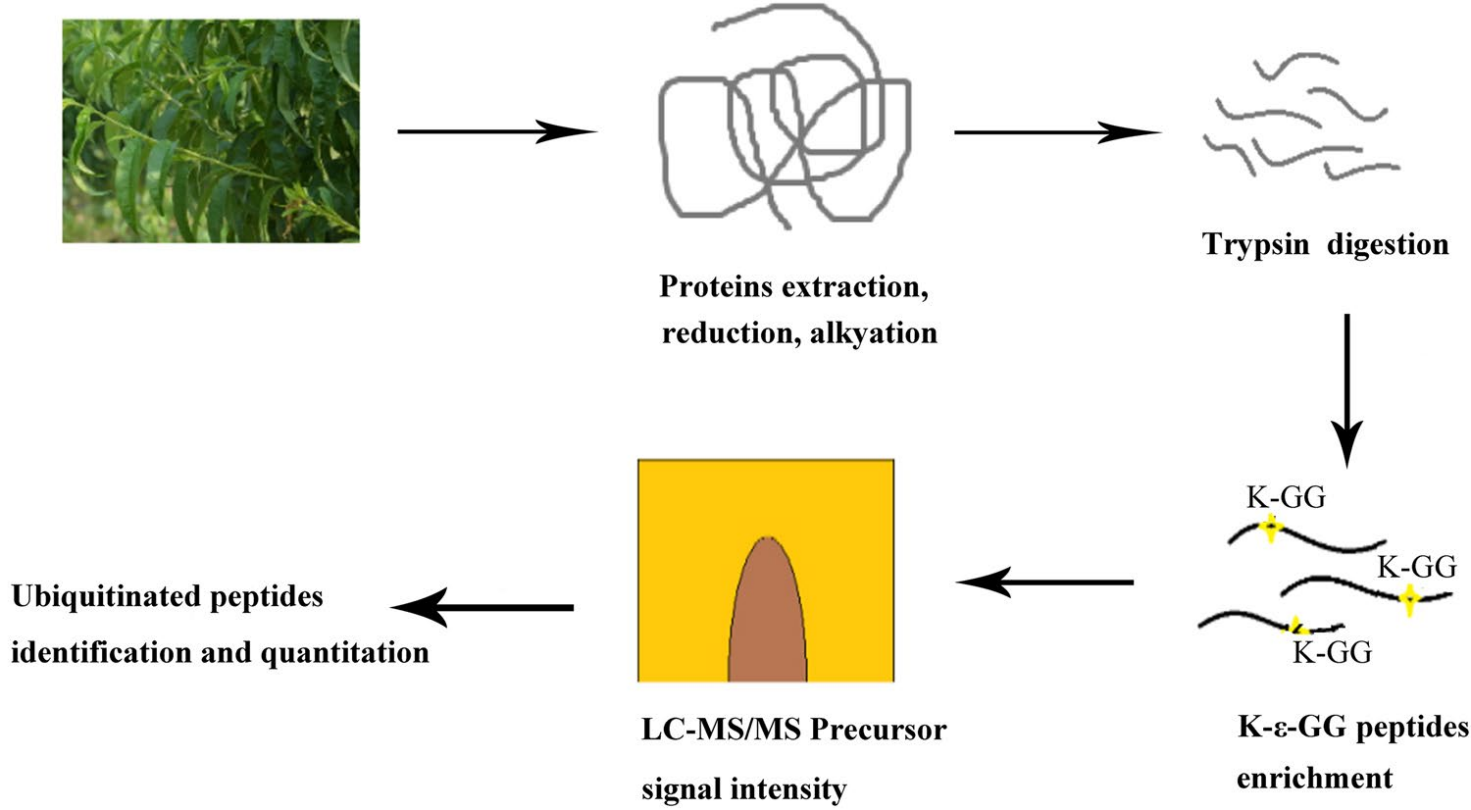
Proteomics-based analysis of ubiquitin modification sites in proteins from peach leaves. Total protein is extracted from peach leaves, reduced by dithiothreitol, subjected to alkylation with iodoacetamide, and then digested by trypsin. Peptide fragments that contain the diglycine remnant motif characteristic of ubiquitin modification are captured by anti-K-ε-GG antibodies. The enriched ubiquitinated fragments are then separated by LC and analyzed by tandem MS/MS.

**Figure 2:**
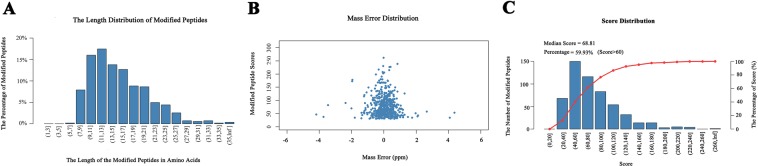
Proteomic identification of ubiquitinated peptides in peach. (**A**) Length distribution of the detected diglycine-modified peptides. The peptides are grouped based on the number of amino acids that they contain. (**B**) Mass error distribution of all detected diglycine-modified peptides. The x axis indicates the calculated mass error for each peptide, whereas the y axis denotes the Andromeda score that represents the probability of a peptide-spectrum match. (**C**) Andromeda analysis of the detected diglycine-modified peptides. The peptides are grouped based on their Andromeda scores, which represent the probability of confident peptide-spectrum matches. The number of peptide fragments that belong to each score category is shown by the column chart with the primary y-axis, whereas the line chart with the secondary y axis denotes the cumulative percentage of peptides with an Andromeda score no greater than the specified upper limit of each score category.

The overall proteome-wide analytic approach that we employed for the identification of lysine modification sites and ubiquitinated protein targets was illustrated in Fig. 1. After digesting the proteins extracted from Okubo peach leaves with trypsin, ubiquitin-conjugated peptide fragments were enriched by IAP using antibodies that selectively recognize lysine residues carrying a diglycine-modified ε-amino group. These peptides were then separated by LC and subjected to tandem MS/MS analysis. In total, the analysis revealed 544 ubiquitination sites on 507 peptide fragments that represented 352 proteins based on the Uniprot *Prunus persica* proteome (Supplementary Table S1). It is worth noting that 94 of these ubiquitination sites, representing 17% of the total, have not been reported previously based on the Swiss-Prot annotation of post-translational modifications (Supplementary Table S2). The overwhelming majority 97.24% of the identified peptide fragments consisted of 8 to 27 amino acids, with an average length of 26.4 (Fig. 2A). All peptide mass measurements achieved a precision of 10 ppm or better (Fig. 2B). Furthermore, Andromeda analysis of the MS2 spectra showed that 59.93% of all detected diglycine-modified peptides yielded a score above 60, with an overall average score of 68.81 (Fig. 2C). Combined, these results confirmed that the MS/MS data that we obtained were of satisfactory quality.

### Analysis of ubiquitination sites and motifs

**Figure 3:**
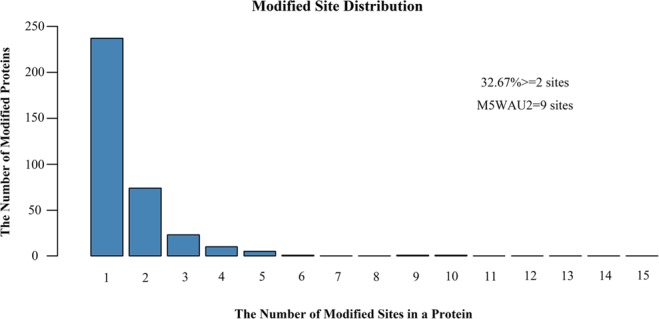
The number of modified sites in the detected diglycine-modified peptides.

We next evaluated the number of ubiquitin modification sites in the protein substrates that we identified. As shown in (Fig. 3), 237 proteins, representing 63.33% of the ubiquitome, contained only one ubiquitination site. Additionally, 74, 23 and 10 of the 352 protein substrates contained two, three and four ubiquitination sites, respectively. In particular, M5WAU2, a 152-amino-acid protein sharing significant homology to ubiquitin and NEDD8, could be ubiquitinated at nine locations. Overall, ubiquitin conjugation site occurs at a frequency of 0.91 per 100 amino acids in all identified protein targets.

**Figure 4:**
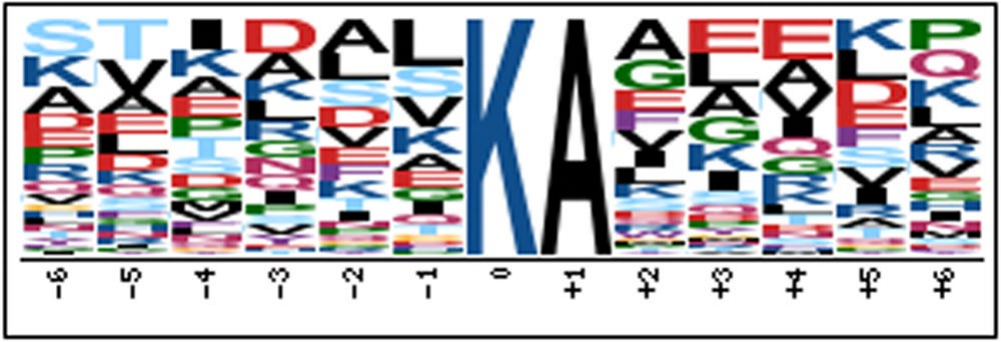
Sequence variation around the identified ubiquitin modification sites. The numbers below the solid line represent the position relative to the diglycine-modified lysine residue (set to 0). The height of each letter denotes the frequency of the corresponding amino acid in the specified position.

To further understand the structural context that promotes ubiquitination in peach, we examined and summarized the common flanking amino acid sequences around the modified lysine residues (Fig. 4). The results showed that 59 peptides featured an alanine at the +1 position relative to the diglycine-modified lysine residue. In comparison, the other positions in the range of −6 to +6 exhibited considerable sequence variability.

### Functional analysis of the ubiquitinated proteins

**Figure 5:**
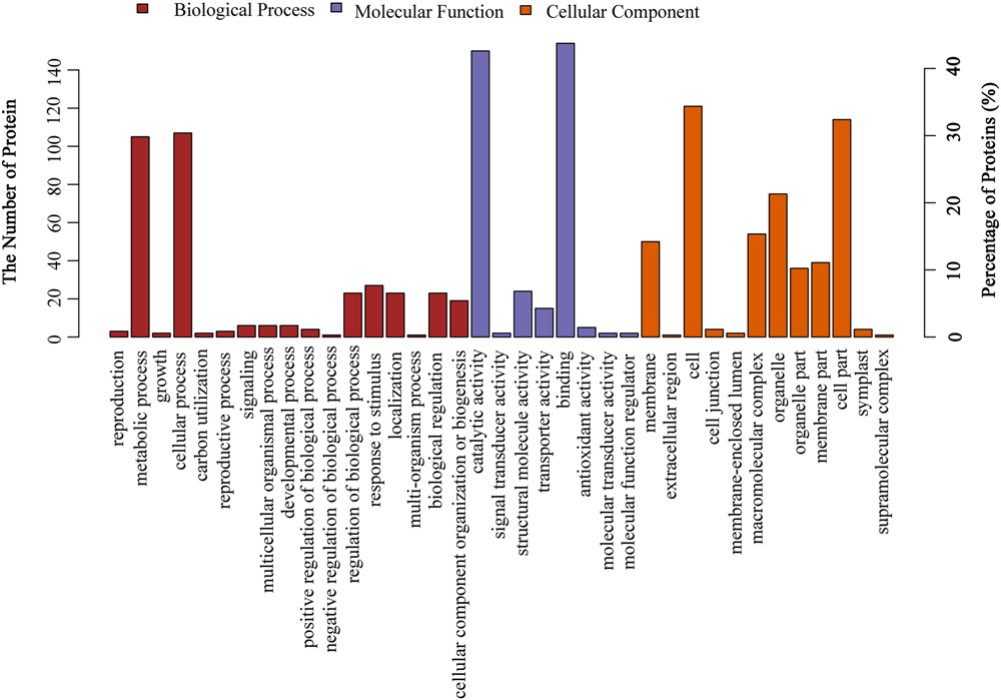
GO enrichment analysis of the ubiquitinated protein substrates. The number of proteins enriched to each GO term in one of the three subcategories, including biological process, molecular function and cellular component, is shown (E-value filter, 1e-6; annotation cut-off, 75).

We next conducted functional analysis of all ubiquitinated proteins that we identified from our LC-MS/MS study. GO enrichment analysis showed that the protein substrates targeted for ubiquitination are predominantly involved in metabolic process or cellular process under the subcategory of biological process (Fig. 5). In addition, most of the modified proteins are associated with molecular functions such as catalytic activity and binding (Fig. 5). This is hardly surprising as these proteins often possess important regulatory and/or metabolic roles that necessitate precise spatial and temporal control. Moreover, cellular components analysis suggested that the identified candidates mostly resided in cytosol, with a substantial portion either present in organelles or as a part of macromolecular complexes (Fig. 5).

**Figure 6:**
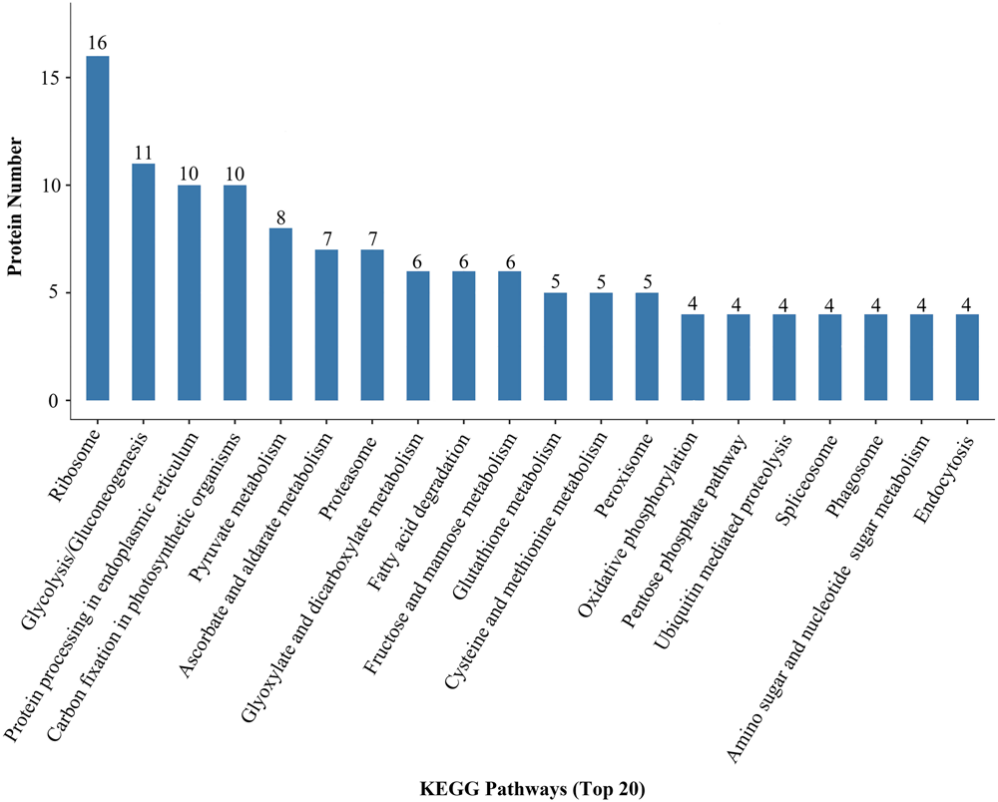
KEGG pathway analysis of the ubiquitinated protein substrate. The KEGG pathways are arranged in descending order by the number of proteins (represented by the number on each column and the y-axis). Only the top 20 KEGG pathways are included.

On the other hand, the KEGG enrichment analysis successfully attributed 352 protein candidates to a total of 69 pathways. As illustrated in Fig. 6, ribosome is the most enriched KEGG pathway, followed by glycolysis/gluconeogenesis, protein processing in endoplasmic reticulum, and carbon fixation in photosynthetic organisms. In addition, the ubiquitinated proteins were also found to be involved in the metabolism of glutathione, fatty acids and amino acids such as cysteine and methionine, as well as other essential cellular processes such as vesicular trafficking (Fig. 6). These findings reinforced the well-established notion that essential biosynthetic and metabolic activities in peach and other species are tightly regulated by ubiquitination.

### Analysis of the whole leaf proteome

**Figure 7:**
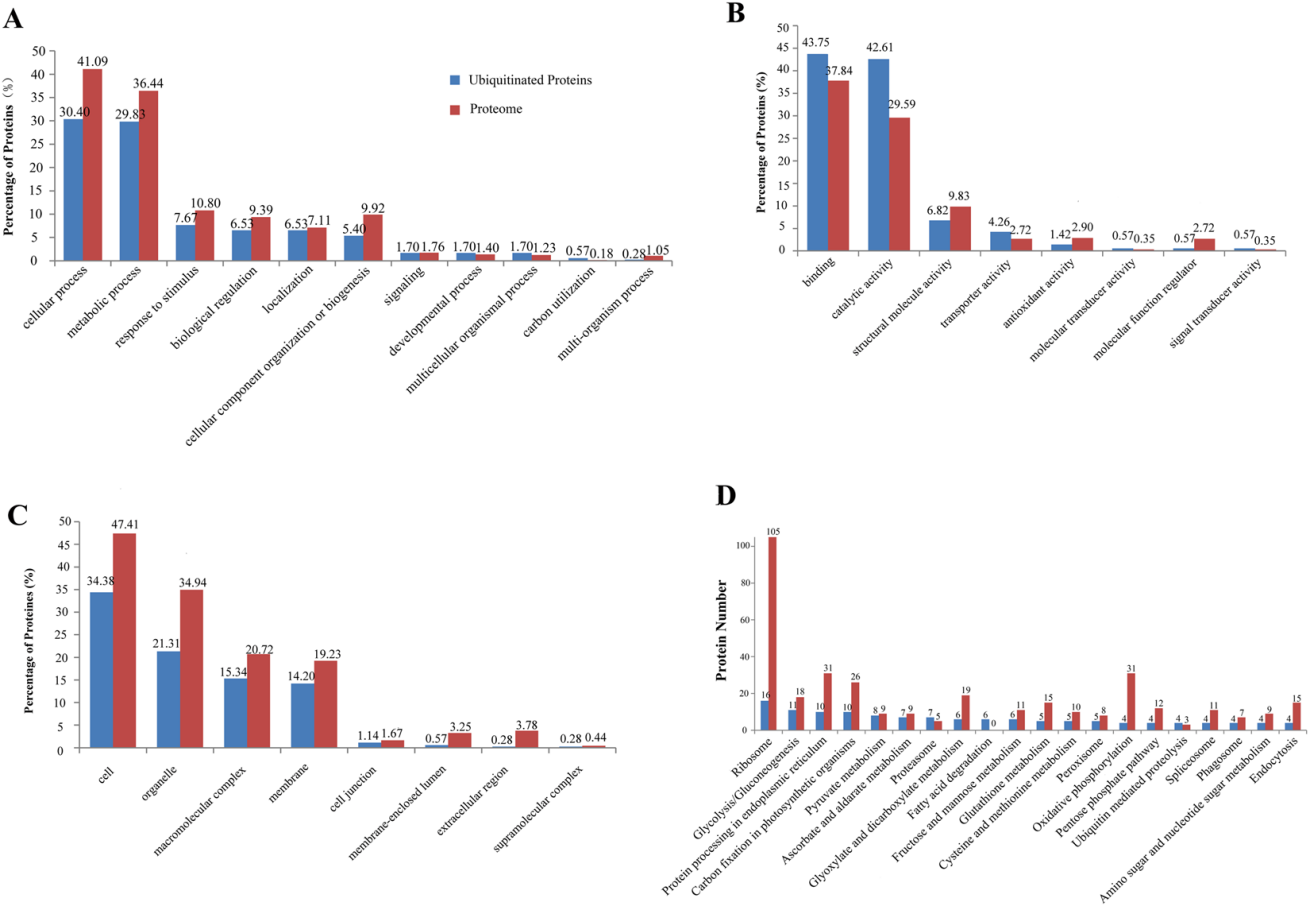
GO classification analysis and KEGG pathway of ubiquitinated proteins compared to global proteome. (**A**) biological process, (**B**) molecular function, (**C**) cellular component and (**D**) KEGG pathway (Top 20).

To gain further understanding of the potential roles and significance of ubiquitination in peach leaves, we also analyzed the leaf proteome. We identified a total number of 1,139 proteins (Supplementary Table S3). A comparison between the members of the global proteome and those of the ubiquitome showed a partial overlap between the two. We speculated that this discrepancy was likely because the enrichment with anti-K-ε-GG antibodies greatly facilitated the capture of low-abundance proteins. The GO and KEGG pathway annotations of the leaf proteome were summarize in (Fig. 7). Similar to the results we obtained for the ubiquitinated leaf proteins, the main GO functions associated with the leaf proteome included cellular process and metabolic process under the subcategory of metabolic process, as well as binding and catalytic activity under the subcategory of molecular functions. The most significant KEGG pathways included those related to ribosome, protein processing in endoplasmic reticulum, carbon fixation in photosynthetic organisms, glyoxylate and dicarboxylate metabolism, as well as glycolysis/gluconeogenesis.

## Discussion

In our current study, we extracted total proteins from ground ‘Okubo’ peach leaves, from which we then selectively enriched ubiquitinated peptide fragments with antibodies that specifically recognized and bound to lysine residues with a diglycine-modified ε-amino group. LC-MS/MS analysis of these fragments revealed a total of 352 proteins carrying 544 ubiquitination sites. To better understand the functional relevance of ubiquitination, we also profiled the global proteome of peach leaves and identified a total of 1,139 proteins. A comparison of our results to those reported in a number of other studies suggested that the size of the peach leaf proteome is similar to those of other plants^20,21^. However, it should be emphasized that the identified leaf proteome showed only a partial overlap with the aforementioned ubiquitome. This is likely because the ubiquitinated proteins were enriched by anti-K-ε-GG antibodies prior to their separation and identification, which allowed the capture of low-abundance members. Xie *et al*. profiled the proteome and ubiquitome of tea leaves, and identified 4,789 and 781 members, respectively^22^, with a similar partial overlap between the two. It should be noted that their proteomic study was conducted on samples subjected to drought stress, which might have stimulated the expression, and therefore enabled the subsequent detection, of many low-abundance proteins, leading to an increase in the leaf proteome size. Functional analysis of all identified proteins suggested that ubiquitination played a key role in regulating a wide range of physiological and cellular processes in peach, particularly carbon metabolism, translation, vesicular trafficking and histone assembly. These findings constitute the first comprehensive and systematic analysis of ubiquitinated proteins in peach.

The increasing awareness that ubiquitination functions as a critical post-translational protein modification mechanism has prompted vigorous efforts to identify all ubiquitinated proteins and map their lysine modification sites, sometimes collectively referred to as the ubiquitome, in different species. Such information would greatly improve our understanding of the global dynamics of UPS and its regulatory roles in various physiological processes. In addition, these ubiquitination substrates and their cognate E3 ligases might also serve as attractive therapeutic targets^23,24^. However, there are several factors that render proteome-wide ubiquitination analysis a daunting challenge. Unlike E1s and E2s, most species possess several hundreds of E3s that interact with an even greater number of structurally and functionally diverse target proteins. Moreover, E3-substrate interactions are transient and often lead to rapid target degradation. In recent years, substrate binding domains in different E3 families, as well as various proteasomal subunits, have been structurally characterized by X-ray crystallography^25–27^ revealing a number of different degradation signals that can be used to systematically identify ubiquitinated protein targets^28^. Nevertheless, many established E3 substrates lack such a motif sequence^29^ suggesting either the existence of as-yet-unknown degrons or interaction mechanisms.

Earlier searches for ubiquitin-modified proteins were usually limited to substrates of a single E3 ligase or those that undergo ubiquitination under specific conditions. For instance, a screening method that compared the degradation patterns between proteins treated with mitotic *Xenopuslaevis* egg extracts, which contained the activated anaphase-promoting complex/cyclosome (APC/C) E3 ligase, and those with interphase extracts, revealed geminin as a substrate targeted by ubiquitination during cell division^30,31^. A more systematic approach involves the use of ubiquitin conjugated to an affinity tag to facilitate the enrichment, purification and detection of modified proteins. This was excellently demonstrated in Sato *et al*.’s use of FLAG-labeled ubiquitin to identify targets of the heterodimeric E3 ligase BRCA1-BARD1 (breast cancer 1, early onset BRCA1 associated RING domain)^32^. However, to overcome the generally weak E3-substrate interaction, overexpression of the ubiquitin tag and the catalytic ligase is required^33^. Moreover, the presence of the affinity tag could affect the ubiquitination process and cause sterically sensitive substrates to be underrepresented in the results^33^. Walton and colleagues systematically mapped ubiquitin-modified lysine residues in Arabidopsis thaliana cell cultures using ubiquitin combined fractional diagonal chromatography and identified 1,607 target substrates carrying a total of 3,009 sites^34^. Recently, the advent of diglycine remnant affinity proteomics has enabled the high-throughput proteomic screening of ubiquitinated substrates in a theoretically unbiased manner. Using this method, Ponts and coworkers obtained the first *Plasmodium falciparum* ubiquitome and found that enhanced ubiquitination activities correlated with the trophozoite stage and schizont stage of the parasite erythrocytic cycle, suggesting that UPS is involved in modulating the morphological development of the protozoan^35^. Additionally, Zhang *et al*. discovered 433 distinct diglycine-modified lysine residues on a total of 285 proteins from wheat seedlings^18^. Impressively, Kim *et al*.’s comprehensive analysis of human ubiquitome based on the same method unearthed 19,000 ubiquitination sites on 5,000 proteins^36^. This is echoed by Sebastian *et al*.’s report of 11,054 putative diglycine-modified lysine residues in a total of 4,273 human proteins^37^. Despite these achievements and the success that we demonstrated in the current study, it should be emphasized that a potential drawback of employing diglycine-specific antibodies is its inability to recognize whether the detected remnant motifs resulted from modification with ubiquitin or some other ubiquitin-like proteins^38,39^. It has been proposed that combining the use of anti-K-ε-GG antibodies with affinity tag-labeled ubiquitin could mitigate this problem^33^.

**Figure 8:**
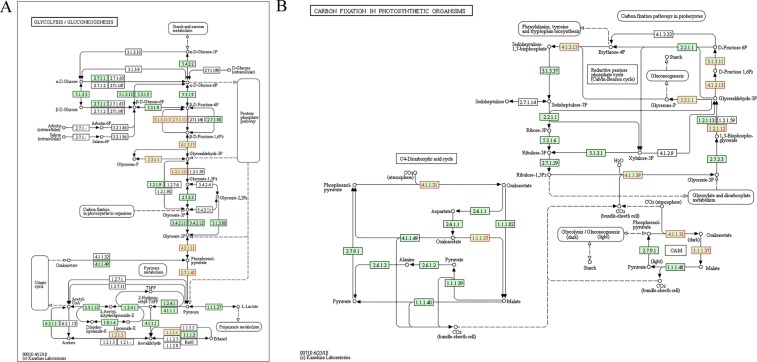
Ubiquitinated proteins that participate in glycolysis/gluconeogenesis and related metabolic pathways in peach. EC. numbers representing the ubiquitinated proteins that we identified in our current study are colored in red as follows: 2.7.1.40, M5W5K0 and M5WMV6; 1.2.1.3, M5XBG8; 4.1.2.13, M5VYU7 and M5W2H9; 4.1.1.39 E3W0K1 and M5XB14; 2.7.1.11, M5XC66; 1.2.1.12, M5XQ59; 1.1.1.37, M5Y9C1; 5.3.1.1 M5X2A0; 4.2.1.11 M5VPR4; 1.1.1.1 M5VJ94. Source: Kanehisa *et al*.^62^.

The results of our current study suggested that ubiquitination plays a key role in regulating a wide array of metabolic pathways in peach. We identified a total of 15 ubiquitinated enzymes involved in glycolysis/gluconeogenesis and/or photosynthesis, including two pyruvate kinases (M5W5K0 and M5WMV6, EC. 2.7.1.40), two aldehyde dehydrogenases (A0A251RFC2 and M5XBG8, EC. 1.2.1.3), two fructose-biphosphate aldolases (M5VYU7 and M5W2H9, EC. 4.1.2.13), two ribulose bisphosphate carboxylases (large chain, RUBISCO, E3W0K1 and M5XB14, EC. 4.1.1.39), one phosphoglycerate kinase (M5X0L9, EC. 2.7.2.3), one 6-phosphofructokinase (M5XC66, EC. 2.7.1.11), one glyceraldehyde-3-phosphate dehydrogenase (M5XQ59, EC. 1.2.1.12), one malate dehydrogenase (M5Y9C1, EC. 1.1.1.37), one triose-phosphate isomerase (M5X2A0, EC. 5.3.1.1),one enolase (M5VPR4, EC. 4.2.1.11), and one alcohol dehydrogenase (M5VJ94, EC. 1.1.1.1) (Fig. 8A,B, Supplementary Table S4). Ubiquitin-dependent degradation of glycolytic enzymes has been previously shown to exert strong modulatory effects on plantand animal physiology. Zhang *et al*. reported that phosphorylation-activated ubiquitination of cytosolic pyruvate kinase 6 in cotton might be associated with accelerated fiber elongation^40^. In another study, Almeida and colleagues showed that APC/C-induced degradation of 6-phosphofructokinase significantly suppressed glycolysis, and thus proliferation, in tumor cells^41^. Additionally, our experimental data indicated that other metabolic processes, including but not limited to cysteine and methionine metabolism, pentose phosphate pathway, glutathione metabolism, glycerolipid metabolism, arachidonic acid metabolism, as well as starch and sucrose metabolism, are also regulated by ubiquitin modification. Once again, some of these ubiquitinated substrates have been confirmed elsewhere, such as sucrose synthase^42^ and phospholipase D^43^. Overall, the pool of metabolic proteins that we found ubiquitinated in the current study showed significant overlap with those reported for wheat^19^, rice^18^ and *Arabidopsis thaliana*^44^. Combined, these results highlighted the critical role of ubiquitination in regulating plant metabolism and development.

Our proteomic investigation also revealed that UPS is involved in the regulation of other crucial molecular processes in peach. Not surprisingly, we found 18 diglycine modification sites on a total of 12 histone isoforms and variants, including those of nine H2As (M5VKW3, M5VRK7, M5W1M5, M5W1N0, M5W1R5, M5WDC2, M5X0T3, M5X7W0 and M5Y070), one H2B (M5WQC2), one H3 (M5WKJ0) and one H4 (M5VRN0). These findings echoed several earlier profiling analyses that similarly indicated extensive ubiquitination of histone proteins^18,45,46^. More importantly, as shown in (Supplementary Table S5), the majority of these ubiquitinated lysine residues were revealed to be located at the C-terminal histone tails, known to play a critical role in nucleosome assembly and thus gene expression^47–49^. Numerous studies have pointed out that histone ubiquitination is a crucial regulatory mechanism that controls both higher-order chromatin structure and gene transcription^50,51^. For instance, it has been described that H2B mono ubiquitination is triggered in *Arabidopsis thaliana* exposed to *Verticillium dahlia* toxins as an important defense mechanism, leading to enhanced microtubular depolymerization through the up-regulation of protein tyrosine phosphatases^52^. On the other hand, polyubiquitination-initiated proteasomal degradation of H3 serves as a prerequisite to chromatin condensation during spermatozoa maturation in rat testes^53^. Ribosomal proteins comprised the second largest group of non-metabolic proteins that we identified as ubiquitination substrates, which included 40 S ribosomal protein s4 (A0A251NKH8), s12 (A0A251NH70 and M5XB76), s24 (M5WAW2), s30 (M5W5G9) and s3a (M5Y710), as well as 60S ribosomal protein l13 (M5X2A2), l19 (M5W1D3) and P0 (M5XF09). These post-translational modification events have been described in detail in *Arabidopsis thaliana*^54^, rice^18^, as well as wheat^19^, and are known to be a part of the ribosome quality control machinery^55,56^. We also showed that two proteasomal subunits (M5VQX2 and M5WUT5) and one proteasome endopeptidase complex (M5XZ63) were ubiquitinated. Besche *et al*. previously reported that autoubiquitination of proteasomal regulatory subunit Rpn 13 could inhibit the association between proteasome and ubiquitin-labeled substrates, suggesting that it could serve as a strategy to prevent undesirable proteolysis in the event of UPS dysfunction^57^. Nevertheless, due to the moderate selectivity and complexity of ubiquitination, there is also speculation that proteasomal autoubiquitination might simply be the result of nonspecific ubiquitin transfer from the modified substrates^19^. Taken together, the above findings lent convincing evidence to the indispensability of ubiquitination for fundamental cellular processes such as gene transcription, translation, protein homeostasis, etc.

## Conclusion

In summary, our study revealed that UPS is tightly involved in regulating a wide array of molecular and physiological processes in peach, mirroring an array of similar ubiquitin profiling studies conducted in various other biological systems. We are convinced that the results that we have obtained could pave the way for future research on how ubiquitination contributes to the agricultural traits of different peach cultivars and other crop species.

## Methods and Materials

### Preparation of protein extracts

We chose *Prunus persica* L. Batch ‘Okubo’, one of the most popular and widely cultivated peach cultivars in China, for proteomic analysis. Specifically, we selected nine well-grown two-year-old Okubo peach trees at the experimental peach orchard of the Institute of Pomology, Shanxi Academy of Agricultural Sciences. From each tree, we then randomly selected and removed four similarly sized, young but fully unfolded healthy leaves. The leaves of all trees were then mixed together. The leaves were then immediately frozen in liquid nitrogen and stored at −80 °C.

Protein extraction was performed following a previously described protocol with minor modifications^58^. Roughly 3 g of peach leaves were first frozen in liquid nitrogen and then ground with a pestle and a mortar. The resultant paste was mixed with 5 vol of pre-chilled extraction solvent consisting of 10% (v/v) trichloroacetic acid and 90% (v/v) acetone. The suspension was allowed to stand at −20 °C for 4 h and subsequently centrifuged at 6000 g, 4 °C for 40 min. After discarding the supernatant, the pellet was washed with pre-chilled acetone three times and air-dried. Approximately 20–30 mg of the dried extracts were resuspended in 30 vol of SDT lysis buffer consisting of 4% (w/v) SDS, 100 mM Tris-HCl at pH 7.6 and 1 mM dithiothreitol, boiled for 15 min, and then centrifuged at 14,000 g for 40 min. The supernatant was passed through a 0.22 µm filter (Millipore, USA) and stored at −80 °C.

Total protein content in each prepared supernatant was measured by the Bradford method on a Multiskan GO Microplate UV is Spectrophotometer (Thermo Fisher Scientific, USA) and visualized by electrophoresis on a 12.5% sodium dodecylsulfate polyacrylamide gel (SDS-PAGE) at a constant current of 14 mA for 90 min, followed by Coomassie blue staining (Supplementary Fig. S1).

### Trypsin digestion

For trypsin digestion, dithiothreitol was added to each sample containing 200 μg protein to a final concentration of 10 mM, followed by incubation in a thermomixer at 37 °C and 600 rpm for 2 h. The sample was then cooled to room temperature and reacted with 50 mM of iodoacetamide in the dark for 30 min. The resultant reaction mixture was diluted in 40 μL of 25 mM NH_4_HCO_3_ buffer, and trypsin was added to an enzyme/substrate weight ratio of 1: 50. After overnight digestion at 37 °C, peptides were obtained by adding trifluoroacetic acid (TFA) to a final concentration of 1%. The supernatant was collected and desalted through an Empore C18 SPE Cartridge (7 mm inner diameter, 3 mL volume, Sigma-Aldrich, USA). The flow-through was lyophilized and reconstituted in 40 µL of 0.1% (v/v) formic acid. Total peptide content was estimated by measuring the absorbance at 280 nm and using extinction coefficients of tryptophan as well as tyrosine for calculation.

### Enrichment of ubiquitin-modified peptides

Lyophilized peptides were re-dissolved in 1.4 mL of pre-chilled immunoaffinity purification (IAP) buffer consisting of 50 mM 3-(N-morpholino) propanesulfonic acid at pH 7.2, 10 mM Na_2_HPO_4_ and 50 mM NaCl. The reconstituted solution was incubatedat 4 °C for 2 h with 250 μg of anti-K-ε-GG antibodies crosslinked on agarose beads from PTMS can Ubiquitin Remnant Motif Kit (Cell Signal Technology, USA), followed by centrifugation at 2,000 g for 30 s. The pelleted beads were washed with 1 mL of pre-chilled IAP buffer twice and with 1 mL of pre-chilled, sterilized ultrapure water for another three times, followed by elution with 0.15% TFA. The eluted peptides were lyophilized and stored at −80 °C. For analysis of the leaf proteome, the peptides obtained after trypsin digestion were directed separated without enrichment.

### LC-MS/MS analysis

Peptide separation was conducted using an EASY-nLC1000 Liquid Chromatograph (Thermo Fisher Scientific). Briefly, 1–2 μg of the enriched ubiquitinated peptides were reconstituted in solvent A (0.1% (v/v) formic acid in water) and then loaded onto a 100 μm × 2 cm Acclaim PepMap 100 NanoViper C18 column (Thermo Fisher Scientific, USA) connected to a 75 μm × 10 cm EASY C18-A2 analytical column (3 μm resin, Thermo Fisher Scientific, USA). The columns were pre-equilibrated with solvent A and the peptides were separated at a constant flow rate of 300 nL/min with a linear gradient of solvent B (0.1% (v/v) formic acid and 84% (v/v) acetonitrile in water) as follows: 0–55% B over 220 min, then 55–100% B over 8 min, and finally 100% B for 12 min.

The eluted peptides were analyzed on a Q Exactive mass spectrometer (Thermo Fisher Scientific, USA) under positive-ion mode. The ions were first subjected to one survey scan in the m/z range of 300–1,800 with mass resolution of 70,000 at 200 m/z, automatic gain control target of 1 × 10^6^, dynamic exclusion at 60 s and maximum IT at 50 ms, followed by 20 data-dependent high-energy collisional dissociation MS2 scans with normalized collision energy of 30 eV, mass resolution of 17,500 at 200 m/z, underfill ratio of 0.1%, isolation window at 2 m/z and maximum ion injection time of 10 ms.

### Protein identification and quantitation

The proteins and ubiquitination sites were identified using MaxQuant software (version 1.3.0.5, Max Planck Institute of Biochemistry, Martinsried, Germany)^59,60^. The tandem MS data obtained were searched against the *Prunus persica* Database (released on Jul 25, 2018, downloaded from https://www.uniprot.org/), which contains 54,093 annotated proteins. The hits were then queried against a reverse decoy database to remove artifacts and possible contaminants. The precursor mass tolerance was set at 6 ppm, and the MS/MS tolerance was set at 0.5 Da. Trypsin/P was specified as the enzyme, and two missed cleavages were permitted. During the database search, the fixed modification was carbamido methylation for cysteines, the variable modifications were Gly-Gly modification for lysines, the false discovery rate (FDR) threshold for modification site was 0.01, the minimum peptide length was 5, and a MaxQuant score was set at ≥20.

### Identification of ubiquitination motifs

The 13-mer sequence flanking the modified lysine residue (from −6 to +6) in each identified ubiquitinated protein were entered as queries into Motif-X at http://motif-x.med.harvard.edu., with the Uniprot Prunus persica reference proteome as background database. Occurrence and significance were set to 20 and 0.00018 (corresponding to Benferroni corrected p value of 0.05), respectively. All other parameters were set to default values. Only overrepresented sequence patterns with statistical significance exceeding the preset threshold would be displayed by the software.

### Functional analysis

Gene Ontology (GO) annotation was performed via the Gene Ontology Database at http://www.geneontology.org using Blast2GO (version 3.3.5)^61^. Briefly, The identified ubiquitinated protein sequences were locally searched against NCBI nr using the NCBI BLAST + client software (ncbi-blast-2.2.28 + -win32.exe) to find homologous proteins from which the functional annotation was transferred to the targeted proteins. Here, the top 10 blast hits with an Evalue less than 1e-3 for each query proteins were retrieved and loaded into Blast2GO for Gene Ontology (GO) mapping and annotation. In this work, an annotation configuration with an E-value filter of 1e-6, default gradual EC weights, a GO weight of 5, and an annotation cutoff of 75 were chosen. The GO functions of the identified ubiquitinated proteins were classified into three subcategories, including biological process, molecular function and cellular process. Kyoto Encyclopedia of Genes and Genomes (KEGG) annotation was performed using the KEGG Automatic Annotation Server at https://www.genome.jp/kegg/kaas. Enrichment analysis was performed using Fisher’s exact test.

## Supplementary information

Supplementary Table S3.

Supplementary Information

.Supplementary Table S1
