# Author Correction: Proteome-wide identification and functional analysis of ubiquitinated proteins in peach leaves

**DOI:** 10.1038/s41598-020-70048-4

**Published:** 2020-07-30

**Authors:** Yanbo Song, Xiaojing Shi, Yanli Zou, Juanru Guo, Nan Huo, Shuangjian Chen, Chengping Zhao, Hong Li, Guoliang Wu, Yong Peng

**Affiliations:** 1 Horticulture College, Shanxi Agricultural University, Taigu, 030801 Shanxi People’s Republic of China; 2 Life Science College, Shanxi Agricultural University, Taigu, 030801 Shanxi People’s Republic of China; 3 Institute of Pomology, Shanxi Academy of Agricultural Sciences, Taigu, 030801 Shanxi People’s Republic of China; 4 Shanghai Applied Protein Technology Co., Ltd, Shanghai, 201100 People’s Republic of China

Correction to: *Scientific Reports* 10.1038/s41598-020-59342-3, published online 12 February 2020

The original version of this Article contained errors in the affiliation list.

Yanbo Song was incorrectly affiliated with ‘Life Science College, Shanxi Agricultural University, Taigu, Shanxi, 030801, PR China’.

Furthermore, Guoliang Wu was incorrectly affiliated with ‘Horticulture College, Henan Agricultural University, Zhengzhou, Henan, 450002, PR China’.

The correct author affiliations are listed below.

Affiliation 1

Horticulture College, Shanxi Agricultural University, Taigu, Shanxi, 030801, PR China.

Yanbo Song, Guoliang Wu

Affiliation 2

Life Science College, Shanxi Agricultural University, Taigu, Shanxi, 030801, PR China.

Xiaojing Shi, Yanli Zou, Juanru Guo, Nan Huo, Chengping Zhao and Hong Li

Affiliation 3

Institute of Pomology, Shanxi Academy of Agricultural Sciences, Taigu, Shanxi, 030801, PR China.

Shuangjian Chen

Affiliation 4

Shanghai Applied Protein Technology Co., Ltd, Shanghai, 201100, PR China.

Yong Peng

These errors have now been corrected in the PDF and HTML versions of the article.

