# Supplementary material for: Proteome-wide identification and functional analysis of ubiquitinated proteins in peach leaves
Source: Sci Rep. 2020 Feb 12;10:2447. doi: 10.1038/s41598-020-59342-3 (PMC7015887; doi:10.1038/s41598-020-59342-3)
Supplement: Supplementary file 2 — Supplementary Information [file 41598_2020_59342_MOESM2_ESM.pdf]

Supplementary Information for

Proteome-wide identification and functional analysis of ubiquitinated proteins in peach leaves

Yanbo Song[a], Xiaojing Shi[a], Yanli Zou[a], Juanru Guo[a], Nan Huo[a], Shuangjian Chen[b], Chengping Zhao[a], Hong Li[a], Guoliang Wu[c], Yong Peng[d]

[a] Life Science College, Shanxi Agricultural University, Taigu Shanxi 030801, PR China

[b] Institute of Pomology, Shanxi Academy of Agricultural Sciences, Taigu Shanxi 030801, PR China

[c] Horticulture College, Henan Agricultural University, Zhengzhou Henan 450002, PR China*

[d] Shanghai Applied Protein Technology Co., Ltd, Shanghai 201100, PR China

* Corresponding author:

Guoliang Wu

Horticulture College, Henan Agricultural University, 63 Nongye Road, Zhengzhou Henan, 450002, PR China

Tel.: +86 139 3909 2535

Fax: +86 371 6355 5339

E-mail address: walnut-wu@126.com (G. Wu)

Table of contents

	Page
**Supplementary Figure S1**	-3-
**Supplementary Table S1**	-4-
**Supplementary Table S2**	-5-
**Supplementary Table S3**	-9-
**Supplementary Table S4**	-10-
**Supplementary Table S5**	-11-

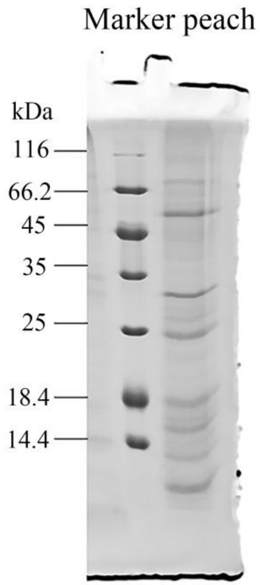

Supplemtary Figure S1. SDS-PAGE gel picture.

Supplementary Table S1

Provided as a separate excel file.

Supplementary Table S2. List of unique peptides containing novel ubiquitination sites.

Protein	Sequence length	Positions within proteins	Sequence window	GlyGly (K) Probabilities	Score	Mass error [ppm]
A0A251MXH4	531	269	PVMGMRTTRSVNLNQKNQFPGLETRDSVLKY	SVNLNQK(1)NQFPGLETR	116.43	0.3619
A0A251MXH4	531	380	STDNGEREVKTQRITKGSFVKSKSPKVKTAG	ITK(0.999)GSFVK(0.001)	69.825	-0.27
A0A251NIC1	284	5	___________MVLEKSLSSAINTKIMGSSG	VLEK(1)SLSSAINTK	123.28	-0.331
A0A251NKX3	242	10	______MGKSFKDSLKALEADIQHANTVALD	DSLK(1)ALEADIQHANTVALDYPR	127.83	-0.269
A0A251NVY5	330	198	EQVDWIGQKNQIDAAKAAGVKQIVLVGSMGG	NQIDAAK(0.991)AAGVK(0.009)	42.976	-0.555
A0A251NVY5	330	317	AFDLASKPEGTGTPTKDFKALFSQITTRF__	AFDLASK(0.009)PEGTGTPTK(0.821)DFK(0.17)	33.308	0.829
A0A251P6V1	495	173	AREEQWKIAVAELSHKLVHATRKRDEAQLEA	IAVAELSHK(1)LVHATR	80.534	-0.513
A0A251P7D8	597	299	RGQGENVGPSEMGASKAAEVELEKLTAPNGR	GQGENVGPSEMGASK(1)AAEVELEK	60.278	-0.413
A0A251PD87	378	221	SKELLDSDSSQCAVCKDLFELGEEAKQMPCK	ELLDSDSSQCAVCK(0.997)DLFELGEEAK(0.003)	31.775	-0.41
A0A251PD87	378	194	LAENDPNRYGTPPASKSAVEGLPVVKISKEL	YGTPPASK(1)SAVEGLPVVK	83.418	0.0606
A0A251PD87	378	231	QCAVCKDLFELGEEAKQMPCKHIYHSDCILP	DLFELGEEAK(0.998)QMPCK(0.002)	63.185	0.0612
A0A251PFU0	867	150	REVYLKCHHCMKEEKKTIVSCSKCKKNSYCV	EEK(1)K(1)TIVSCSK(0.001)	35.652	0.2487
A0A251PFU0	867	149	NREVYLKCHHCMKEEKKTIVSCSKCKKNSYC	EEK(1)K(1)TIVSCSK(0.001)	35.652	0.2487
A0A251PTF6	395	140	AICHGLLVLAPAGAVKGRKCTAVSTMKPGLV	KPIAAICHGLLVLAPAGAVK(1)GR	36.856	0.5206
A0A251Q5J9	443	241	ARFGQNVNKSRVLPTKCDDLGRDQREMIGKL	VLPTK(1)CDDLGRDQR	56.225	0.4941
A0A251Q5J9	443	221	EFIDGVVMKRHGLSLKAVKNARFGQNVNKSR	HGLSLK(0.999)AVK(0.001)	83.206	0.1179
A0A251QGC1	501	233	QDDDDDFDRALSLSLKTAEQEKAIREEKRKD	ALSLSLK(0.98)TAEQEK(0.02)	52.555	0.2225
A0A251QI71	509	318	RIPMEAEQKTVLVQQKTIDHPKSRDLRESLK	TVLVQQK(1)TIDHPK	116.01	0.0735
A0A251QI71	509	311	SDQTEQPRIPMEAEQKTVLVQQKTIDHPKSR	IPMEAEQK(1)TVLVQQK	62.042	-0.517
A0A251QL49	315	257	PGGPSAPLDYSNYDQKPKSGKMGLGTGLAVG	QAGYSGPGGPGGPGGPSAPLDYSNYDQK(0.803)PK(0.197<br>)	36.202	-0.271
A0A251QPT0	159	51	QQHFVVSLPIPKKETKMAVTKQVPEVEEEKL	K(1)ETK(1)MAVTK	41.448	0.0007
A0A251QPT0	159	48	HTLQQHFVVSLPIPKKETKMAVTKQVPEVEE	K(1)ETK(1)MAVTK	41.448	0.0007
A0A251R5G9	1026	147	MANNASELERLRNLVKELEEREVKLEGELLE	NLVK(1)ELEER	81.338	-0.465
M5VJ62	401	222	GAKVTKCRSTRLVNMKQVSPRVPAGNYPVAH	LVNMK(1)QVSPR	72.006	-0.255
M5VJL5	555	44	KFSVRASLDSTVGDFKAILAQNCDIPAEQQR	ASLDSTVGDFK(1)AILAQNCDIPAEQQR	35.94	1.0112
M5VM21	419	270	RMGPTSESEISPETLKRIQRVKKLTKMSEDV	MGPTSESEISPETLK(1)R	36.638	0.8015
M5VQ60	361	6	__________MASAQKDLPAAGEKPVSTENK	ASAQK(0.928)DLPAAGEK(0.071)PVSTENK(0.001)	37.157	0.0872

M5VQ60	361	284	EGRTALHFACGYGEVKCAQVLLEAGARVDAL	TALHFACGYGEVK(1)CAQVLLEAGAR	45.011	0.0776
M5VQ60	361	87	LLNDPSIKELAEQIAKDPSFNQMADQLQKTF	ELAEQIAK(1)DPSFNQMADQLQK	88.176	-0.095
M5VRA2	164	14	__MVLKTELCRFSGAKIYPGKGIRFIRSDSQ	FSGAK(1)IYPGK	117.02	-0.458
M5VRC4	171	165	SESEKLRMKKLEELSKNVDSLQ_________	KLEELSK(1)NVDSLQ	117.92	0.2165
M5VRE7	163	58	FQKRIEEEEHLLAAIKAAARIRALKLSEEDY	RIEEEEHLLAAIK(1)AAAR	58.671	0.1521
M5VRE8	146	136	ILTQLKKEMASPHNRKLVQPPEGTYF_____	K(1)LVQPPEGTYF	78.655	0.6218
M5W072	322	314	ARDENLAKKLWDFSNKLIDGASKS_______	LWDFSNK(1)LIDGASKS	66.285	-0.106
M5W072	322	97	RSARVDVLTLDLCSIKSVRAFVDSFNALDLP	VDVLTLDLCSIK(1)SVR	45.33	-0.217
M5W0H8	163	11	_____MSLVTAEIKSKADELYHGDGICQEKS	SK(1)ADELYHGDGICQEK	94.297	0.4334
M5W1L0	158	152	LSVTVPKAEVKKPDVKAIEISG_________	K(0.061)PDVK(0.939)AIEISG	59.198	0.4584
M5W204	442	94	QSNPENTVGVLTMAGKGVRVLVTPTSDLGKI	TQSNPENTVGVLTMAGK(1)GVR	172.66	-1.968
M5W204	442	159	QQRIIVFAGSTVKHDKKFLEIIGRRLKKNSV	HDK(0.589)K(0.411)FLEIIGR	62.303	-0.313
M5W204	442	160	QRIIVFAGSTVKHDKKFLEIIGRRLKKNSVA	HDK(0.5)K(0.5)FLEIIGR	62.303	0.5814
M5WAU2	154	29	TLEVESSDTIDNVKAKIQDKEGIPPDQQRLI	AK(1)IQDKEGIPPDQQR	120.3	-1.211
M5WAU2	154	33	ESSDTIDNVKAKIQDKEGIPPDQQRLIFAGK	IQDK(1)EGIPPDQQR	46.408	-4.187
M5WAU2	154	63	KQLEDGRTLADYNIQKESTLHLVLRLRGGAK; KQLEDGRTLADYNIQKESTLHLVLRLRGGTM	TLADYNIQK(1)ESTLHLVLR	216.99	-0.321
M5WAU2	154	11	_____MQIFVKTLTGKTITLEVESSDTIDNV	TLTGK(1)TITLEVESSDTIDNVKAK	226.44	0.345
M5WAU2	154	6	__________MQIFVKTLTGKTITLEVESSD	MQIFVK(1)TLTGK	156.51	-0.502
M5WAU2	154	87	RLRGGTMIKVKTLTGKEIEIDIEPNDTIDRI	TLTGK(1)EIEIDIEPNDTIDR	117.29	0.1849
M5WAU2	154	27	TITLEVESSDTIDNVKAKIQDKEGIPPDQQR	TITLEVESSDTIDNVK(0.5)AK(0.5)	60.209	0.0305
M5WAU2	154	124	KEGIPPVQQRLIYAGKQLGDDKTAKDYNIEG	LIYAGK(1)QLGDDKTAK	214.65	0.3332
M5WAU2	154	48	KEGIPPDQQRLIFAGKQLEDGRTLADYNIQK	LIFAGK(1)QLEDGRTLADYNIQK	261.65	-0.016
M5WB03	426	279	KGKIPCATCGSRGLIKCLTCQGSGSLLTRNV	GLIK(1)CLTCQGSGSLLTR	65.716	-0.146
M5WB62	395	52	ISECKPKAKIVDLCEKGDSYIREQTGNMYKN	IVDLCEK(1)GDSYIR	51.875	0.7268
M5WBC5	376	62	LTNVLPRGQKLICKGKLLVDGMTLKESEVAN	GK(1)LLVDGMTLK	96.229	-0.031
M5WDV8	171	39	RDMVSVITNRVTDLHKSGSVNQEDDDETGVR	VTDLHK(1)SGSVNQEDDDETGVR	92.31	-0.042
M5WHX7	184	5	___________MAMTKPHFQAEQNAIIEQKQ	AMTK(1)PHFQAEQNAIIEQK	45.876	0.7109
M5WIK8	354	158	QLENGVARLDHPPASKAAIESMPVIKIADDH	LDHPPASK(0.999)AAIESMPVIK(0.001)	35.176	-0.152
M5WNR2	158	43	KMQDILQCSGEDVQSKYARFKVEKDVNLPSL	MQDILQCSGEDVQSK(1)YAR	40.085	-0.182
M5WNR2	158	11	_____MAELPKFIALKSFTSGKYLVYKDQAT	FIALK(0.999)SFTSGK(0.001)	68.069	0.506

M5WQZ9	1208	803	NAEVIPPPRLGDDYLKGGFNENLAQRQNNEI	LGDDYLK(1)GGFNENLAQR	51.147	-0.588
M5WQZ9	1208	740	IEIRKDIDELLDLSQKLRDQREQFIKERESF	KDIDELLDLSQK(1)LR	70.98	-1.143
M5WSH1	423	206	AAQGPIDPHQPSSSSKSFTGTARLLSGETVP	QLAAAQGPIDPHQPSSSSK(1)SFTGTAR	41.084	0.4412
M5WSH1	423	266	RLDDPENAPFLESIKKSECPKELEPANRRTA	RLDDPENAPFLESIK(0.463)K(0.537)	67.605	0.2609
M5WSU3	159	68	VNQPSLVHMKSTYSDKYLRTASEDSPWIVAE	STYSDK(1)YLR	55.567	-0.854
M5WSU3	159	145	SGTDFEDDALAAANAKPTSTPAFTVEKLPG_	SGTDFEDDALAAANAK(0.772)PTSTPAFTVEK(0.228)LPG	58.272	0.3852
M5WT59	321	187	ITNRHLRSDSHCPVCKDKFELGSEARQMPCN	SDSHCPVCK(0.863)DK(0.137)FELGSEAR	63.337	0.0734
M5X0S9	183	78	MKGDLGIGSVREVNVKSGLPATTSTERLELL	EVNVK(1)SGLPATTSTER	52.823	0.4437
M5X0S9	183	163	DETCYFVEALIRCNLKSLADVSERMAVQDRT	CNLK(1)SLADVSER	52.555	-1.043
M5X0U7	331	115	GKDVKEAIVKEIPTAKIDAIELDLSSLSSVR	EIPTAK(1)IDAIELDLSSLSSVR	85.737	0.5054
M5X0W5	323	235	KGNKYVLGEFLEFKGKQEDVEALRNIKRSKI	GK(1)QEDVEALR	59.542	0.2014
M5X0W5	323	257	LRNIKRSKISRLIVQKTSMLGFAPSRLQVLY	LIVQK(1)TSMLGFAPSR	54.598	0.2368
M5X1C0	312	310	PDAPKRSYEDLFGSIKQR______________	SYEDLFGSIK(1)QR	130.01	-1.071
M5X2G7	207	160	DSTIGPVEGLALNLVKEQQSKRRYTDTANFT	DSTIGPVEGLALNLVK(0.994)EQQSK(0.006)	79.568	-0.031
M5X2H3	183	92	RNYLNLPSEIVPATLKKQAKPPGRPLGPSGD	NYLNLPSEIVPATLK(0.5)K(0.5)	79.81	0.2345
M5X2H3	183	93	NYLNLPSEIVPATLKKQAKPPGRPLGPSGDR	NYLNLPSEIVPATLK(0.5)K(0.5)	79.81	0.2345
M5X2H3	183	142	GYRSGPRAPGGDFGDKGGAPADYRPSFGGSR	APGGDFGDK(1)GGAPADYR	30.974	-0.271
M5X2T5	159	56	HTLVAKVLTSNPVLQKGRSVSQHLRQTRISE	VLTSNPVLQK(1)GR	100.72	0.4938
M5X6D3	1116	262	SFELMRKEQQKAFQEKQKLKPEKNKGDFDFA	AFQEK(1)QK(1)LK(1)	41.876	-0.204
M5X6D3	1116	266	MRKEQQKAFQEKQKLKPEKNKGDFDFATLLD	AFQEK(1)QK(1)LK(1)	41.876	-0.204
M5X6D3	1116	264	ELMRKEQQKAFQEKQKLKPEKNKGDFDFATL	AFQEK(1)QK(1)LK(1)	41.876	-0.204
M5X7B6	448	24	GAQASKGQGKDIAVSKAQMGESKSVPSNPTK	DIAVSK(0.813)AQMGESK(0.188)SVPSNPTK(0.999)	30.094	0.6427
M5X7B6	448	39	KAQMGESKSVPSNPTKKMRFTSSFEKDPSST	DIAVSK(0.813)AQMGESK(0.188)SVPSNPTK(0.999)	30.094	0.6427
M5XBV2	441	145	LFGQFFSALERNLYFKTLPDGNDDPVRLDKA	NLYFK(1)TLPDGNDDPVR	63.894	0.1696
M5XCW1	333	98	VVSTKIFWGGPGPNDKGLSRKHVVEGTKASL	IFWGGPGPNDK(1)GLSR	64.394	-0.121
M5XFV6	514	8	________MEFFNTAKAVKLRSHLDKYLVAD	MEFFNTAK(0.821)AVK(0.179)	45.879	-0.315
M5XH45	164	71	VNRLIETLSTPSPLSKRYGTLSADEASAAAR	LIETLSTPSPLSK(1)R	75.462	-0.166
M5XHN6	136	5	___________MAESKPGLRKPVFTKVAQLR	AESK(1)PGLR	94.302	0.3037
M5XKE4	953	123	KEFGKSMDELFLDFVKVPLATASIAQVHRAT	SMDELFLDFVK(1)VPLATASIAQVHR	45.395	0.0176
M5XKT5	197	60	DVKGVDDTKALAVVEKAPETEVKKPSGGSID	ALAVVEK(1)APETEVK	62.14	-0.326
M5XKT5	197	47	AEEKAVVPPPREVDVKGVDDTKALAVVEKAP	EVDVK(1)GVDDTK	67.08	0.073

M5XKT5	197	53	VPPPREVDVKGVDDTKALAVVEKAPETEVKK	GVDDTK(1)ALAVVEKAPETEVK	146.78	0.0747
M5XM44	159	150	LAQPPEGNEDGRGDQKGLVVKCCIF______	LAQPPEGNEDGRGDQK(0.968)GLVVK(0.032)	44.587	0.7269
M5XM44	159	72	TVHEGRICQLKLFCGKDYPDNPPTVRFQTRI	LFCGK(1)DYPDNPPTVR	67.136	0.2042
M5XMY7	232	218	VKDVMPETEKLAEVAKIITAKLRGAGAPPK_	LAEVAK(1)IITAK	105.03	0.1295
M5XPR5	537	176	ETKPTHEPKIEENPLKEAEEVAKEATQLAVP	IEENPLK(0.999)EAEEVAK(0.001)	49.31	0.0756
M5Y3G7	650	255	QEVERAIVACEGDLQKAAESLRASKQDPPSV	AIVACEGDLQK(1)AAESLR	134.85	-0.476

Supplementary Table S3
Provided as a separate excel file.

Supplementary Table S4. Ubiquitinated peptides involved in pathways of Carbon fixation in photosynthetic organisms and Glycolysis / Gluconeogenesis in peach.

Protein	KEGG Pathway	Description	EC number
M5VX90	Glycolysis / Gluconeogenesis	fructose-1,6-bisphosphatase	EC:3.1.3.11
M5XC66	Glycolysis / Gluconeogenesis	6-phosphofructokinase	EC:2.7.1.11
M5W2H9	Glycolysis / Gluconeogenesis	fructose-bisphosphate aldolase, class I	EC:4.1.2.13
M5VYU7	Glycolysis / Gluconeogenesis	fructose-bisphosphate aldolase, class I	EC:4.1.2.13
M5X2A0	Glycolysis / Gluconeogenesis	triosephosphate isomerase (TIM)	EC:5.3.1.1
M5XQ59	Glycolysis / Gluconeogenesis	glyceraldehyde 3-phosphate dehydrogenase	EC:1.2.1.12
M5VPR4	Glycolysis / Gluconeogenesis	enolase	EC:4.2.1.11
M5W5K0	Glycolysis / Gluconeogenesis	pyruvate kinase	EC:2.7.1.40
M5WMV6	Glycolysis / Gluconeogenesis	pyruvate kinase	EC:2.7.1.40
M5XBG8	Glycolysis / Gluconeogenesis	aldehyde dehydrogenase (NAD+)	EC:1.2.1.3
M5VJ94	Glycolysis / Gluconeogenesis	alcohol dehydrogenase class-P	EC:1.1.1.1
E3W0K1	Carbon fixation in photosynthetic organisms	ribulose-bisphosphate carboxylase large chain	EC:4.1.1.39
M5VX90	Carbon fixation in photosynthetic organisms	fructose-1,6-bisphosphatase I	EC:3.1.3.11
M5VYU7	Carbon fixation in photosynthetic organisms	fructose-bisphosphate aldolase, class I	EC:4.1.2.13
M5W2H9	Carbon fixation in photosynthetic organisms	fructose-bisphosphate aldolase, class I	EC:4.1.2.13
M5WNQ0	Carbon fixation in photosynthetic organisms	phosphoenolpyruvate carboxylase	EC:4.1.1.31
M5X2A0	Carbon fixation in photosynthetic organisms	triosephosphate isomerase (TIM)	EC:5.3.1.1
M5XAV2	Carbon fixation in photosynthetic organisms	phosphoenolpyruvate carboxylase	EC:4.1.1.31
M5XB14	Carbon fixation in photosynthetic organisms	ribulose-bisphosphate carboxylase small chain	EC:4.1.1.39
M5XQ59	Carbon fixation in photosynthetic organisms	glyceraldehyde 3-phosphate dehydrogenase	EC:1.2.1.12
M5Y9C1	Carbon fixation in photosynthetic organisms	malate dehydrogenase	EC:1.1.1.37

Supplementary Table S5. Summary of identified ubiquitinated histones and the locations of the modified lysines in peach leaves.

Protein	Sequence length	Positions within proteins	Fasta headers	Sequence window	GlyGly (K) Probabilities	Intensity P
M5VKW3	138	134	>tr|M5VKW3|M5VKW3_PRUPE Histone H2A OS=Prunus persica OX=3760 GN=PRUPE_8G233200 PE=3 SV=1	AGGGVIPHIHKSLINKTSKE___________	SLINK(1)TSKE	7471400000
M5VRK7	135	131	>tr|M5VRK7|M5VRK7_PRUPE Histone H2A OS=Prunus persica OX=3760 GN=PRUPE_7G236800 PE=3 SV=1	AGGGVIPHIHKSLINKSTKE___________	SLINK(1)STKE	908540000
M5VRN0	103	32	>tr|M5VRN0|M5VRN0_PRUPE Histone H4 OS=Prunus persica OX=3760 GN=PRUPE_2G232000 PE=3 SV=1	KRHRKVLRDNIQGITKPAIRRLARRGGVKRI	DNIQGITK(1)PAIR	1563000
M5W1M5	152	128	>tr|M5W1M5|M5W1M5_PRUPE Histone H2A OS=Prunus persica OX=3760 GN=PRUPE_6G250200 PE=3 SV=1	HGGVLPNINPVLLPKKPERVAKKEPKSPAKG	LLAGVTIAHGGVLPNINPVLLPK(0.449)K(0.551)PER	39269000
		127		AHGGVLPNINPVLLPKKPERVAKKEPKSPAK	LLAGVTIAHGGVLPNINPVLLPK(0.778)K(0.222)PER	24357000
M5W1N0	150	127	>tr|M5W1N0|M5W1N0_PRUPE Histone H2A OS=Prunus persica OX=3760 GN=PRUPE_6G082300 PE=3 SV=1	AHGGVLPNINPVLLPKKSEKAAAKEPKSPSK	LLAGVTIAHGGVLPNINPVLLPK(0.761)K(0.187)SEK(0.053)	36719000
		128		HGGVLPNINPVLLPKKSEKAAAKEPKSPSKA	LLAGVTIAHGGVLPNINPVLLPK(0.472)K(0.528)	17897000
		29		RKGGGPKKKPVTRSVKAGLQFPVGRIGRYLK	SVK(1)AGLQFPVGR	14713000
M5W1R5	141	131	>tr|M5W1R5|M5W1R5_PRUPE Histone H2A OS=Prunus persica OX=3760 GN=PRUPE_6G332900 PE=3 SV=1	PNIHQTLLPKKVGKGKGDIGSASQEF_____	GK(1)GDIGSASQEF	8333100
M5WDC2	151	126	>tr|M5WDC2|M5WDC2_PRUPE Histone H2A OS=Prunus persica OX=3760 GN=PRUPE_6G322000 PE=3 SV=1	SGGVLPNINPVLLPKKTSNASSEAAEKAPKS	LLQGVTIASGGVLPNINPVLLPK(0.468)K(0.532)	9702700

M5WKJ0	136	80	>tr|M5WKJ0|M5WKJ0_PRUPE Histone H3 OS=Prunus persica OX=3760 GN=PRUPE_4G054300 PE=3 SV=1	KLPFQRLVREIAQDFKTDLRFQ SHAVLALQE	EIAQDFK(1)TDLR	24801000
		57		FRPGTVALREIRKYQKSTELLIR KLPFQRLV	K(0.497)YQK(0.503)STELLIR	3415800
M5WQC2	134	118	>tr|M5WQC2|M5WQC2_PRUPE Histone H2B OS=Prunus persica OX=3760 GN=PRUPE_6G250100 PE=3 SV=1	EIQTAVRLVLPGELAKHAVSEG TKAVTKFTS	LVLPGELAK(1)HAVSEGTK	58112000
M5X0T3	132	121	>tr|M5X0T3|M5X0T3_PRUPE Histone H2A OS=Prunus persica OX=3760 GN=PRUPE_4G029500 PE=3 SV=1	NGGVMPNIHNMLLPKKTGTGK SGPSDD____	LLGSVTIANGGVMPNIHNMLLPK(0.47 3)K(0.527)	15453000
		121		NGGVMPNIHNLLLPKKAGTSSK NVGGDDDS_	LLGDVTIANGGVMPNIHNLLLPK(0.466)K(0.534)	31214000
M5X7W0	135	22	>tr|M5X7W0|M5X7W0_PRUPE Histone H2A OS=Prunus persica OX=3760 GN=PRUPE_4G200700 PE=3 SV=1	SIGSGAAKKATSRSSKAGLQFP VGRIARFLK	SSK(1)AGLQFPVGR	14206000
		120		ANGGVMPNIHNLLLPKKAGTSS KNVGGDDDS	LLGDVTIANGGVMPNIHNLLLPK(0.817)K(0.149)AGTSSK(0.034)	16598000
M5Y070	136	132	>tr|M5Y070|M5Y070_PRUPE Histone H2A OS=Prunus persica OX=3760 GN=PRUPE_1G472200 PE=3 SV=1	AGGGVIPHIHKSLINKSTKD____ _______	SLINK(1)STKD	1162200000
